# An Unrecognized Cause of Gastro-Intestinal Irritation in a Child Discovered

**Published:** 1883-09-20

**Authors:** G. G. Roy

**Affiliations:** Professor of Materia Medica in Southern Medical College


### AN UNRECOGNIZED CAUSE OF GASTRO-INTESTINAL IRRITATION IN A CHILD DISCOVERED.

By G. G. Roy, M.D.,

**Professor of Materia Medica in Southern Medical College.**

The perplexity of the physician in assigning the *true cause* of many of the severe complaints of children is often very embarrassing. So it has occurred to me—not once, but many times. And this is not to be wondered at—and, I think, by no means evinces a lack of sound discrimination or a criminal want of etiological knowledge of disease on the part of the physician. Fearing our reputation for professional wisdom may suffer criticism at the hands of our patrons, especially the female attendants of our patients, are we not too apt to assign SOME CAUSE for the many changing phenomena of many of the obscure cases we meet with, hoping only to satisfy the inquisitive demands of the friends of the sick. I do not pretend to say that this is, by any means, *the rule*, but that it is often the case I feel confident.

The causes of disease in children, and adults, known and unknown, are so numerous that the intelligent physician is rarely at a loss to ascribe some plausible cause for most diseases; but that we may be mistaken in one of the most common complaints of children at this season is shown by the following case, which came under my observation a few weeks ago:

I was called upon, at my office, by the mother of a child I had delivered some eighteen or twenty months ago, for a prescription for her baby. Her statement was that, late in the night previous, after being put to bed in perfect health, the infant aroused her by its screams, and in a short while begun to vomit; this continued, at intervals, for several hours, when the motions of its bowels became very frequent—threatening, as she thought, an attack of cholera infantum.

Considering it an attack of disordered stomach and bowels from something the child had eaten, I prescribed a mild anodyne astringent, which only gave temporary relief. In a day or two I was called upon again to prescribe, with the statement that the child's bowels seemed to be getting worse, instead of better, and there was more evidence of pain. I requested her to give it two teaspoonfuls of castor oil, with a drop or two of spirits turpentine, in a little whisky toddy, and in the afternoon report its condition.

This was done next day by the father of the child. He reported the baby better, but it had passed from the bowels, after considerable straining, in its last action, *a mouse* nearly full grown—"tail and all". This I doubted—never having heard of such an occurrence—and did not credit it until he brought the mouse to my office preserved in whisky, with the statement from the mother that she saw the baby pass it.

The mouse is still in my office, and has been examined by a number of professional friends, and the case was reported at a meeting of the Atlanta Medical Society, and none of the medical gentlemen had ever seen or read of a similar case.

The mother's theory of this rather unusual case is this: The weather being very warm, the baby was found to rest better on "a pallet," spread upon the floor of the chamber, and this "pallet" was spread with bedding kept during the day in a closet infested with mice; that this mouse was secreted in the folds of the bedding when the pallet was spread at night, and that, during the night, being of a migratory nature, he concluded "to change front" and, in doing this, wandered to the child's mouth, that was open, and, thinking it a snug hole in which to hide himself, entered, but the child, not being accustomed to such a morsel, clutched down upon his lordship's caudal extremity, which gave him a frightened impetus forward, which he could not, or would not, arrest until the child's stomach was reached. Then began his gyrations for release, which produced the screaming and vomiting that occurred.

The mouse, when brought to my office, presented a shrunken and glazed appearance (due chiefly to the maceration in alcohol), but still encased in a very perceptible coating of the child's mucus.

Now this, I know, is a strange story, but a true one. It will be of great interest to medical men, and will put them to thinking; but it will be of greater interest, coupled with dreaded foreboding, to the thousand and one mothers who may hear of this case, and it will put them to *watching* the mouths of their dear little ones, to see if *they* sleep with them wide enough open for a mouse of *any size* to enter while they are asleep.

